# Efficacy of Topical Mesenchymal Stem Cell Therapy in the Treatment of Experimental Dry Eye Syndrome Model

**DOI:** 10.1155/2014/250230

**Published:** 2014-07-17

**Authors:** Emrullah Beyazyıldız, Ferda Alpaslan Pınarlı, Özlem Beyazyıldız, Emine Rümeysa Hekimoğlu, Uğur Acar, Muhammed Necati Demir, Aynur Albayrak, Figen Kaymaz, Güngör Sobacı, Tuncay Delibaşı

**Affiliations:** ^1^Department of Ophthalmology, Samsun Research and Training Hospital, Kıranköy Mevkii, 55100 Samsun, Turkey; ^2^Center of Cell Research and Genetic Diagnosis, Dışkapı Yıldırım Beyazıt Research Hospital, Etlik, 06010 Ankara, Turkey; ^3^Department of Histology and Embryology, Kastamonu Faculty of Medicine, Hacettepe University, 06230 Ankara, Turkey; ^4^Department of Ophthalmology, Kastamonu Faculty of Medicine, Hacettepe University, 06230 Ankara, Turkey; ^5^Department of Ophthalmology, Faculty of Medicine, Yıldırım Beyazıt University, Bilkent, 06800 Ankara, Turkey; ^6^Department of Pathology, Dışkapı Yıldırım Beyazıt Research and Training Hospital, Dışkapı, 06110 Ankara, Turkey; ^7^Department of Histology and Embryology, Faculty of Medicine, Hacettepe University, 06230 Ankara, Turkey; ^8^Department of Ophthalmology, Faculty of Medicine, Hacettepe University, 06230 Ankara, Turkey; ^9^Department of Endocrinology, Kastamonu Faculty of Medicine, Hacettepe University, 06230 Ankara, Turkey

## Abstract

*Purpose*. The current study was set out to address the therapeutic efficacy of topically applied mesenchymal stem cells (MSCs) on dry eye syndrome (DES) induced by benzalkonium chloride (BAC) in rats.* Methods*. Rats were divided into two groups just after establishment of DES. Eye drops containing either bromodeoxyuridine labeled MSCs (*n* = 9) or phosphate buffer solution (*n* = 7) were topically applied once daily for one week. Schirmer test, break-up time score, ocular surface evaluation tests, and corneal inflammatory index scoring tests were applied to all rats at baseline and after treatment. All rats were sacrificed after one week for histological and electron microscopic analysis.* Results*. Mean aqueous tear volume and tear film stability were significantly increased in rats treated with MSCs (*P* < 0.05). Infiltration of bromodeoxyuridine labeled MSCs into the meibomian glands and conjunctival epithelium was observed in MSCs treated rats. Increased number of secretory granules and number of goblet cells were observed in MSCs treated rats.* Conclusion*. Topical application of MSCs could be a safe and effective method for the treatment of DES and could potentially be used for further clinical research studies.

## 1. Introduction

Dry eye syndrome (DES) is one of the most common ocular diseases in the world [1]. Although DES is a multifactorial and complex disease, inflammation plays a key role in pathogenesis of the disease. Inflammation, regardless of the cause, has been observed during all stages of DES [2]. Today, treatment strategies for DES include application of artificial tears, therapeutic contact lenses, anti-inflammatory drugs (including corticosteroids and cyclosporin A), and punctal occlusion. Recent studies have focused on treatment of DES using anti-inflammatory therapies and have shown that the additive application of anti-inflammatory drugs enhanced epithelial proliferation and differentiation which could be beneficial in the treatment of these patients [3]. Mesenchymal stem cells (MSCs) can differentiate into a variety of cell types, and recent studies are exploring if the multipotent nature of these cells can be exploited for the treatment of a variety of immune-mediated diseases [4]. MSCs are known to protect cells from injury and can directly promote tissue repair [5–8]. While promoting tissue repair directly, MSCs have also been shown to modulate the immune system and attenuate tissue damage induced by excessive inflammation [4].

Despite the range of treatment options, many patients still suffer from DES and show signs associated with ocular surface changes. New treatment regimens should target the core mechanisms that drive DES to be a permanent cure for DES. Stem cells are known to have regenerative effects, homing characteristics to injury site, and differentiation capabilities to diverse cells and may permanently treat DES. Therefore, in the current study we studied the effects of topical application of MSCs on experimental inflammatory DES model in rats.

## 2. Material and Methods

All experiments in the current study were approved by the local ethical animal studies committee at Dıskapı Yıldırım Beyazıt Research and Training Hospital (approval ID: 2012/17) and all procedures were performed in accordance with the ARVO Statement for the Use of Animals in Ophthalmic and Vision Research at Medical Research Center. Sixteen male Wistar rats (220–250 g) were used for this study. Rats were kept in standard environment (relative humidity 60 ± 10%, temperature 25 ± 1°C, and alternating 12 h light-dark cycles) throughout the study.

### 2.1. Experimental Procedure and Grouping

Only right eye of each rat was used throughout the study. Based on previous studies, the right eye of each rat was topically treated twice a day with 0.2% benzalkonium chloride (BAC) for 7 days [9, 10]. After establishment of DES, eye drops containing either bromodeoxyuridine labeled mesenchymal stem cells (*n* = 9) or phosphate buffer solution (PBS) (*n* = 7) were topically applied once daily for one week. At the end of the study, all rats were sacrificed and the right eye of each animal was enucleated for histological analysis and two of them in each group were analyzed with transmission electron microscopy. All experimental procedures were performed under anesthesia of rats with mixture of ketamine (50 mg/kg) and xylazine (10 mg/kg).

### 2.2. Isolation, Expansion, and Labeling of MSCs and Preparation of Eye Drops

Bone marrow was drawn from the femur and tibia of the same animals and mononuclear cells were separated by the density gradient method [11]. Mononuclear cells were identified using flow cytometry (FACS Calibur TM (Becton, Dickinson and Company, Vancouver, Canada)) after collecting cells from the interphase. Hematopoietic and mesenchymal stem cell determinants (BD Pharmingen, San Diego) were used for identification. Once identified, cells were incubated in MCS medium and were propagated with subcultures after colony-forming unit fibroblasts (CFU-F) became visible. Some MSCs were suspended and identified once more using flow cytometry using bone marrow-derived mesenchymal stem cell surface determinants (CD11b/c (−), CD45 (−), CD90 (+), and CD44 (+)). Once passaged, cells were cultured with 1.5 ml of MSC medium Dulbecco's Modified Eagle's Medium-Low Glucose (DMEM-LG) after suspension with 1.5 ml trypsin ETDA-C solution and aliquoted into sterile 2 ml cryotubes and stored at −80°C. Suspension was thawed by rapid thawing method just two hours before topical application.

To label bone marrow-derived MSCs* in vitro*, 10 *μ*l of bromodeoxyuridine (BrdU) solution (1 mM BrdU in 1X Dulbecco's PBS, BD Pharmingen) was carefully added for each ml of tissue culture medium. The cell culture density was 2 × 10^6^ cells/ml and the treated cells were incubated for 2 hours. For MSCs-treated rats, a single 25 *μ*l drop (1 × 10^5^ MSCs suspended in PBS) was applied daily to the conjunctival sac of rats for one week (*n* = 9). BrdU detection was made by using a BrdU detection Kit (BD Pharmingen) to show the presence of BrdU^+^ cells. Single cell suspensions were stained with 0.4% trypan blue for the quantification of cell viability with the countess automated cell counter (Invitrogen). Viability of cells in PBS was found to be 85%.

### 2.3. Tear Volume Measurements, Ocular Surface Evaluation (Break-Up Time (BUT), Fluorescein Staining, and Rose Bengal Staining), and Corneal Inflammatory Index Scoring

All diagnostic tests were performed before and after one week of BAC application to confirm DES and after one week of therapy with either MSCs or PBS. After experimental procedures, all rats were sacrificed and ocular tissues were carefully dissected and harvested for histological analysis, immunofluorescence staining for BrdU^+^ cells, and electron microscopic analysis. Tear volume measurements: tear volume was measured using the modified Schirmer I test. Briefly, after pulling down the lower eyelid, a strip of Whatman 41 filter paper was placed on the palpebral conjunctiva near the junction of the middle and outer third of the eyelid. At each time point, tear volume of the right eye of each rat was measured three times after the eye had been open for 15 seconds. The average length of the wetted portion of each strip of filter paper was considered as the final measurement. BUTscoring: after dropping 1 *μ*l of sodium fluorescein into the conjunctival sac, BUT was measured as the time lapse between the last blink and the appearance of the first random dry spot appearing in the cornea. Measurement was repeated three times and the mean value of the measurements was considered as the final BUT score. Fluorescein staining scoring: corneal epithelial damage was assessed 90 seconds after applying a 1 *μ*l drop of 1% fluorescein into the conjunctival sac. The cornea was divided into 4 quadrants and each quadrant was evaluated independently using the following scoring system: 0 = no staining, 1 = slightly punctate staining (<30 spots), 2 = punctate staining (>30 spots), but not diffuse, 3 = severe diffuse staining, but no positive plaque, and 4 = positive fluorescein plaque. Scores of four quadrants were added to arrive at a final score (maximum score is 16 points) [12]. Rose Bengal staining: Rose Bengal score was measured using the Van Bijsterveld system fifteen seconds after instilling 1 *μ*l of 1% Rose Bengal into the conjunctival sac [13]. The intensity of conjunctival zones and the cornea was scored, respectively: 1+  = few separated spots, 2+  = many separated spots, and 3+  = confluent spots (maximum score is 9 points). Corneal inflammatory index scoring: corneal inflammatory index scoring was evaluated based on the three parameters: ciliary hyperemia, central corneal edema, and peripheral corneal edema grade [14]. Ciliary hyperemia was graded on the following scale: 0 = no ciliary hyperemia, 1 = present <1 mm, 2 = present between 1 and 2 mm, and 3 = present >2 mm. Both central and peripheral corneal edema were graded as follows: 0 = no central corneal edema, 1 = present with visible iris details, 2 = present without visible iris details, and 3 = present without visible pupil. Each animal received an inflammatory index score by adding the scores of these three parameters and dividing by a factor of 9. Blind grading was performed for all scores by a single physician.

#### 2.3.1. Histological and Transmission Electron Microscopy Analysis

Tissues were fixed in 4% paraformaldehyde and then paraffin. Sections were cut into 5 *μ*m sections using a microtome. Tissues were stained with hematoxylin and eosin (H&E) and examined using light microscopy. Fresh corneal and conjunctival samples were fixed in 2.5% glutaraldehyde solution in phosphate buffer at pH 7.4 for 4 h and postfixed for 1 h in 1% osmium tetroxide solution in 0.1 M phosphate buffer solution. After further postfixation, dehydration, embedding, slicing, and staining procedures, sections were examined in JEOL JEM-1400 electron microscope and photographed by CCD camera (Gatan Inc., Pleasanton, CA, USA).

### 2.4. Statistical Analysis

Statistical analysis was performed using SPSS for Windows 15.0. Data distribution was assessed by Kolmogorov-Smirnov or Shapiro-Wilk test. *P* values that were less than 0.05 were considered as statistically significant. Mann-Whitney *U*, Kruskal Wallis variance analysis, and Wilcoxon signed rank tests were used for the multiple comparisons of ocular surface assessment tests between groups.

## 3. Results

After one-week application of topical 0.2% BAC application DES has been established in both groups and has been confirmed by ocular surface tests. Ocular surface tests were significantly decreased/increased from baseline in both groups (*P* < 0.05).

**Figure 1:**
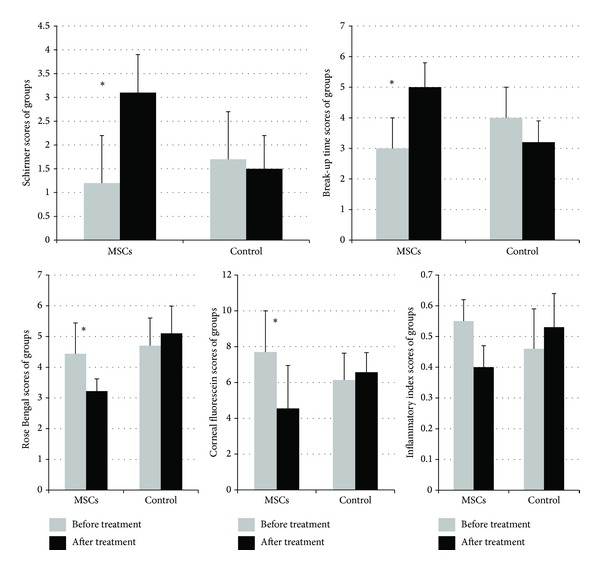
Ocular surface evaluation tests (Schirmer score, break-up time, fluorescein staining, and Rose Bengal staining) and corneal inflammatory index scoring test scores of groups. A marked increase in aqueous tear volume was observed in MSCs-treated rats, while aqueous tear volume decreased in PBS-treated controls after one week of therapy. The mean BUT score increased in MSCs-treated rats, while the score decreased in the PBS-treated group after one week of therapy. The mean Rose Bengal score decreased in MSCs-treated rats, while scores increased in PBS-treated controls. The mean fluorescein score decreased in MSCs-treated rats, while the score increased in PBS-treated controls after one week of therapy. The mean inflammation index scores decreased in MSC-treated rats, while scores increased in PBS-treated controls after one week of therapy. (∗ = *P* < 0.05) within PBS-treated group tear volume and break-up time levels were slightly decreased and Rose Bengal score, the mean fluorescein score, and the mean inflammation index scores were slightly increased with no significant difference (*P* < 0.05) and this might be due to sustained effect of 0.2% BAC or toxicity of diagnostic dyes and repeated manipulations.

**Figure 2:**
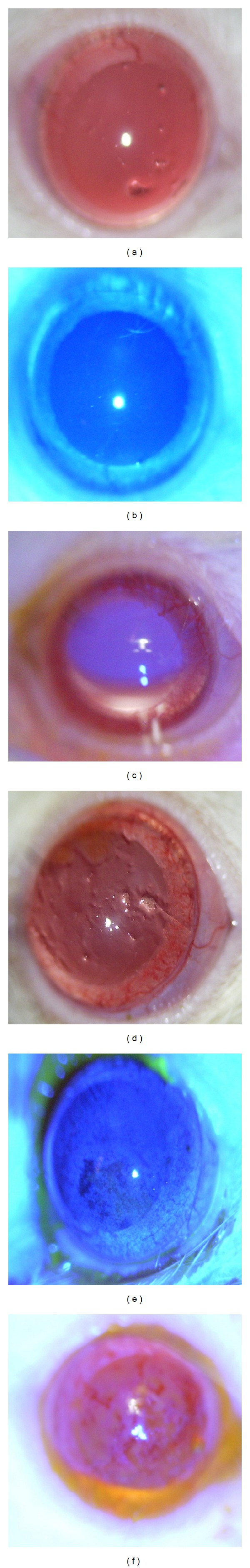
Representative biomicroscopic fluorescein and Rose Bengal staining photographs of groups. MSC-treated group ((a), (b), and (c), resp.) and PBS-treated group ((d), (e), and (f), resp.). Punctate epitheliopathy was observed in PBS-treated rats and there were increased Rose Bengal and corneal fluorescein score grades in PBS-treated rats.

Mean aqueous tear volume and BUT scores of MSCs-treated group significantly increased (*P* = 0.007 and *P* = 0.005, resp.) and were significantly higher than those of PBS-treated group after one week of therapy (*P* = 0.04 and *P* = 0.003, resp.). Corneal epithelial fluorescein and Rose Bengal scores of MSCs-treated group were significantly decreased (*P* = 0.006 and *P* = 0.01, resp.) after one week of therapy. Rose Bengal scores after one week of MSCs therapy were significantly lower than PBS-treated group scores (*P* = 0.001) and corneal fluorescein scores were lower than PBS-treated group scores, not significantly (*P* = 0.07) (Figures 1 and 2).

Although the mean inflammation index scores of MSCs-treated rats were decreased, not significantly (*P* = 0.06), there was a significant difference between groups regarding the mean inflammation index scores after one week of therapy (*P* = 0.02).

**Figure 3:**
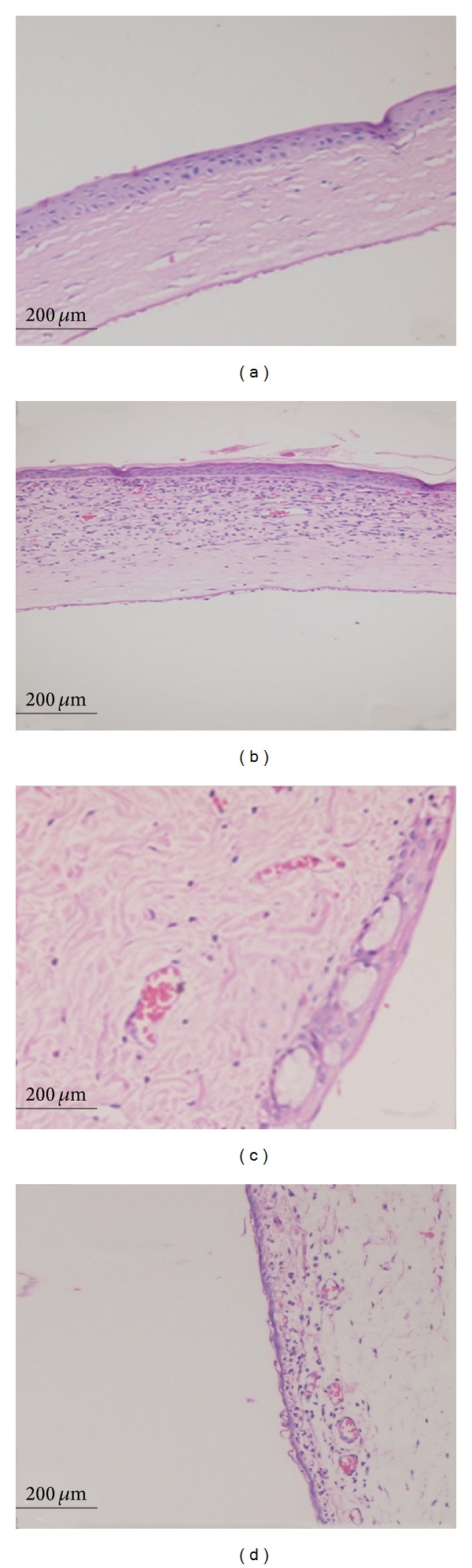
Light microscopy findings. Decreased corneal inflammation was observed in MSCs-treated rat cornea after one week of therapy (a) while diffuse leukocyte infiltration was observed in PBS-treated rat cornea (b). Resolved conjunctival inflammation was observed in MSCs-treated rat conjunctiva after one week of therapy (c) while diffuse leukocyte infiltration and vascular congestion were observed in PBS-treated rat's conjunctiva (d). (H&E staining, magnification: ×200).

There was minimal corneal and conjunctival inflammation in MSCs-treated rats. However, diffuse leukocyte infiltration (primarily, polymorphonuclear leukocyte infiltration) was observed in both the corneas and conjunctiva of PBS-treated rats. In addition, the meibomian glands were also inflamed and congestion of vascular structures was observed in PBS-treated rats (Figure 3).

**Figure 4:**
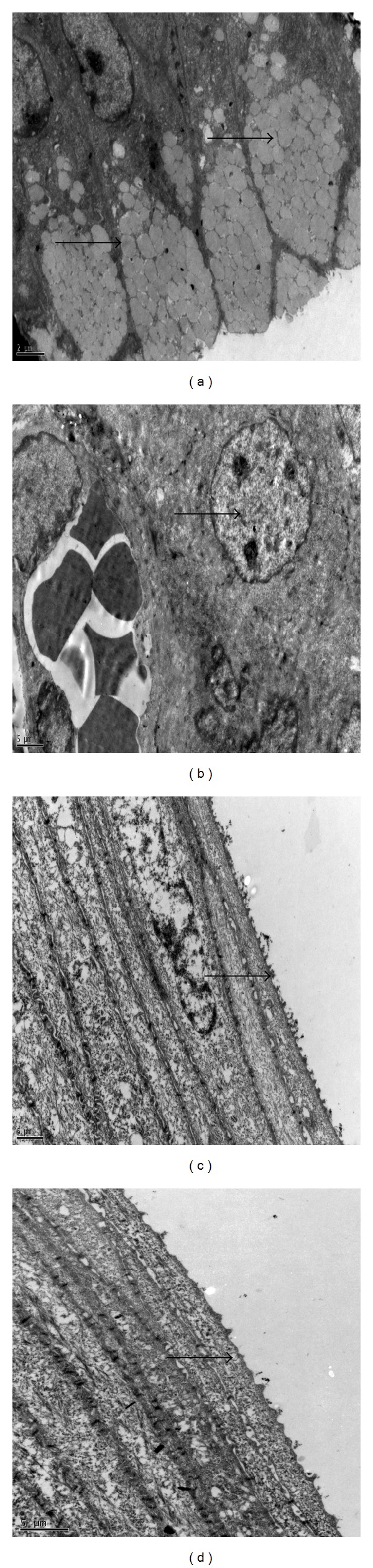
Transmission electron microscopy findings. Increased number of goblet cells and numerous secretory granules were observed in cytoplasm of goblet cells (arrows) in MSCs-treated group after one week of therapy (a). Degenerated goblet cells and decreased secretory granules were observed in cytoplasm of goblet cells (arrows) in PBS group after one week of therapy (b). Microvillus was preserved at apical portions of the corneal epithelium (arrows) of MSCs-treated rats and there was prominent sign of cellular injury at cornea of these rats after one week of therapy (c). Degeneration and loss of microvillus at apical portions of the corneal epithelium and signs of separation of cells from each other were observed in PBS-treated rats (d) (Joel JEM-1400 model, magnification (a), (b): ×8000 and (c), (d): ×12000).

**Figure 5:**
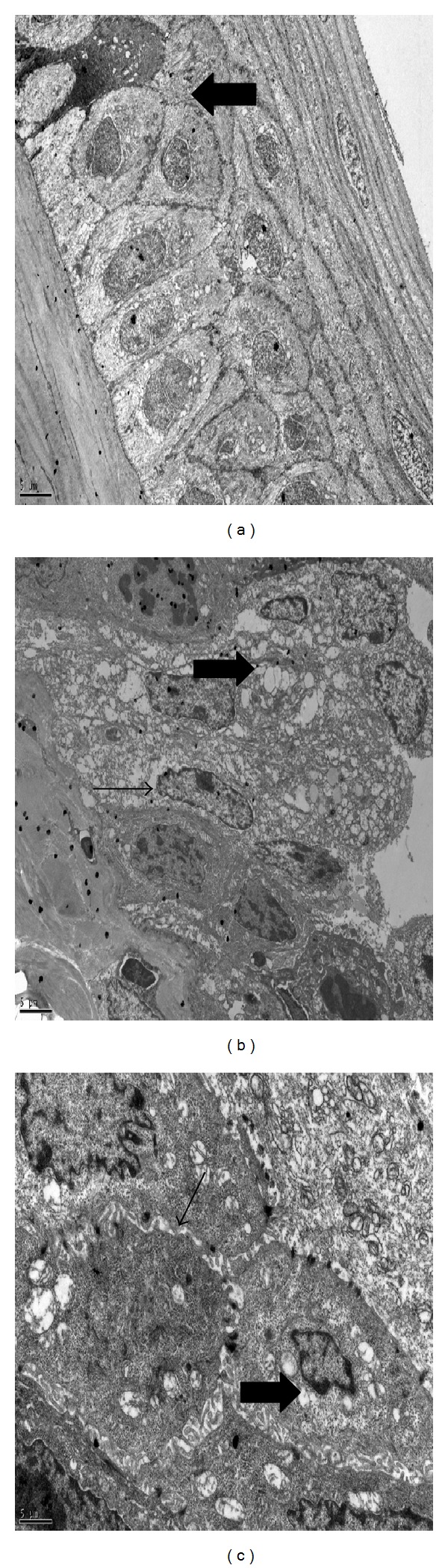
Transmission electron microscopy findings of cellular injury observed in PBS-treated rats. Apoptotic cell (arrows) observed in PBS-treated rat cornea (a). Swelling of cells and lytic focuses in cell cytoplasms (thick arrow) were observed. Condensation of mitochondrial matrix, dilatation of smooth endoplasmic reticulum, and decreased secretory granules (thin arrow) were observed in cells (b). Peripheral migration, condensation of chromatins, and irregular expansion of perinuclear cisternas (thick arrow) were observed in nucleus of cells although there were intact desmosomes (thin arrow) between cells (c) (Joel JEM-1400 model, magnification (a), (b): ×5000 and (c): ×12000).

Decreased number of goblet cells was observed in conjunctiva of PBS-treated rats and number of secretory granules was decreased in goblet cells of these rats. However, there were increased number of goblet cells in conjunctiva of MSCs-treated rats and these cells contained more secretory granules in their cytoplasm. Meanwhile, there was decreased number of microvilli at apical portions of corneal epithelium of PBS-treated rats, and within the corneas of these rats cells started to show signs of separation from each other and mitochondria of these cells were also enlarged. Signs of cellular injury and apoptotic cells were observed at the cornea epithelium in PBS-treated rats. Microvillus was preserved at apical portions of the corneal epithelium of MSCs-treated rats and there was no prominent sign of cellular injury at cornea of these rats (Figures 4 and 5).

**Figure 6:**
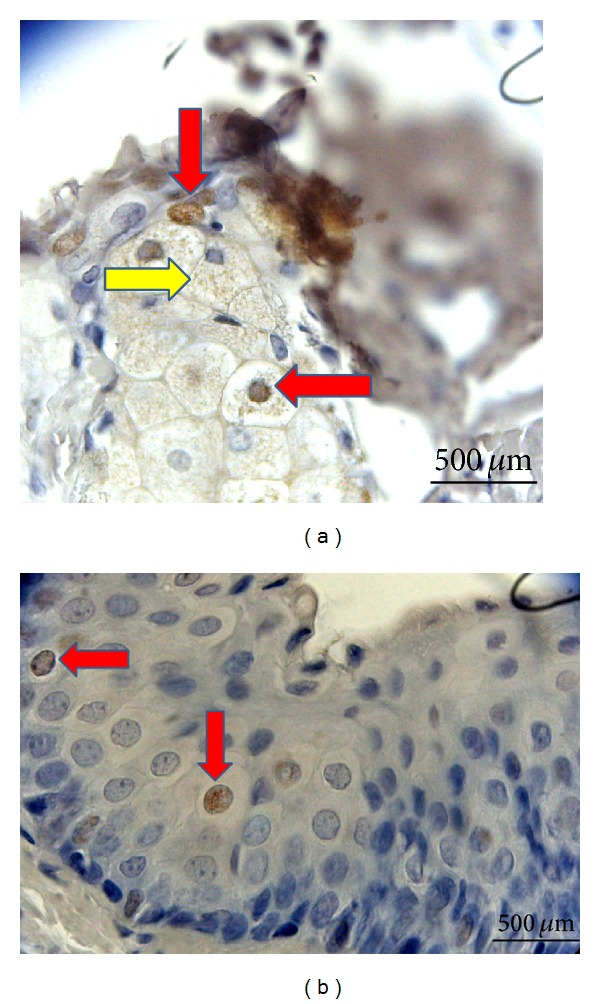
BrdU labeled MSCs were shown to infiltrate the meibomian glands and conjunctival epithelium. (a) Meibomian gland infiltration of BrdU labeled MSCs. Cells with BrdU^+^ stained nucleus (red arrows). Spread of BrdU stained areas to the cytoplasm of some cells (yellow arrow). (b) BrdU^+^ cells in the conjunctival epithelium (red arrow) (Leica HMLB45: Germany, 2000; magnification: ×400).

Conventional method for immunohistochemical staining has been used to quantify number of infiltrated BrdU^+^ cells [15, 16]. Briefly, three areas in three sections in all rats were examined at ×20 magnification and number of counted BrdU^+^ cells to other cells were calculated. The mean result was 6.7% in the treatment group. Topically applied MSCs mostly infiltrated into meibomian glands and conjunctival epithelium. Migration of BrdU labeled MSCs was shown in Figure 6.

## 4. Discussion

The current study shows that topical administration of MSCs could be effective for the treatment of inflammatory type of DES. To the best of our knowledge, this is the first study that evaluated the effect of topical application of MSCs on DES in rats and also showed engraftment of topically applied MSCs into conjunctival epithelium. Inflammation is known to play a major role in any type of DES and perpetuates DES symptoms [2]. In this study, we used BAC induced DES model since it provides a good animal model for inflammatory DES commonly observed in clinics [9, 17, 18] and studied anti-inflammatory effects of topically applied MSCs on this type of DES. MSCs have been shown to have anti-inflammatory effects and have been successfully used to treat a variety of diseases because of their wide ranging differentiation potential, anti-inflammatory, and immunomodulatory effects [19, 20]. Recently, a number of studies have been performed to show anti-inflammatory effect of MSCs on corneal injuries. Ma et al. expanded MSCs on denuded human amniotic membrane and transplanted them onto chemically burned rat corneas. Four weeks after transplantation, they showed that MSCs successfully reconstructed damaged rat corneas and decreased inflammation factors matrix metalloproteinase-2, interleukin-2, and CD45 [21]. This study showed that topically transplanted MSCs on amniotic membrane could ameliorate inflammation and repair corneal injury by their anti-inflammatory effects [21]. In another study, anti-inflammatory effects of subconjunctivally administered MSCs on chemically burned cornea have been researched and it was shown that subconjunctivally administered MSCs decrease CD 68+ macrophage cell infiltration and downregulate mRNA expression levels of macrophage inflammatory protein-1 (MIP-1*α*), tumor necrosis factor-alpha (TNF-*α*), and vascular endothelial growth factor (VEGF) levels and they speculated that decreased macrophage infiltration was due to decreased expression levels of MIP-1*α* [19, 22]. In our study, after one-week topical application of MSC, decreased leukocyte infiltration was observed in cornea and conjunctiva of rats and no prominent inflammatory signs have been observed with histological and ultrastructural analysis. Microvillus structures were preserved at apical portions of the corneal epithelium of MSCs-treated rats and there was no prominent sign of cellular injury at cornea of these rats. These findings demonstrated that topical application of MSCs could decrease inflammation by their anti-inflammatory effects in a BAC induced DES model in rats.

Recently, studies have shown that MSCs could play significant role in corneal epithelial regeneration and transdifferentiate into the corneal epithelial or stromal cells in different type of corneal injury models. Guo et al. have showed migration and differentiation of MSCs into corneal epithelial and stromal cells after being transplanted on surface of chemically burned corneas of rabbits. MSCs transplantation decreased conjunctivalization of cornea and showed regenerative and therapeutic effects on chemically burned rabbit cornea in this study possibly by transdifferentiation into epithelial cells and anti-inflammatory and immunoregulatory effects [23]. In a different study, systemically transplanted MSCs showed engraftment into an injured cornea and promoted wound healing possibly by transdifferentiation and immunomodulatory effects in a chemically burned cornea of rabbit model. They have showed migration of MSCs into injury site and differentiation into myofibroblasts [24]. In the current study, we observed infiltration and homing of BrdU labelled MSCs into the meibomian glands and conjunctiva epithelium. This is the first study in literature that shows migration of BrdU labeled MSCs into conjunctival epithelium and meibomian glands after one-week topical application of MSCs. Together with our study, this work suggests that treatment of DES with MSCs has become a promising approach [3]. In this study, topically applied MSCs effectively treated DES in rats by reducing inflammation and increasing epithelial recovery that was confirmed by histological and ultrastructural analysis. There were increased number of goblet cells and decreased number of meibomian gland injuries in the conjunctiva of MSCs-treated rats. There are limited reports of studies using topical MSCs therapy for other ocular disorders. Gallazzi et al. studied topical effects of BM-derived stem cells (extracted from male mice) on chemically burned corneal injuries in female mice. They analyzed dissected corneas and revealed the presence of male Y-chromosome DNA in the stem cell treated group. Their data showed that topically applied hematopoietic stem cells migrate to sites of injury and integrate with the corneal epithelium [25]. Agorogiannis et al. reported a case of posttraumatic persistent sterile corneal epithelial defect, refractory to standard treatments. They found that topical application of autologous adipose derived MSCs helped the healing process of corneal ulcer [26]. It is important to note that this study was a case report and results were evaluated just for clinical outcome and the migration of the topically administrated AdMSCs was not confirmed histopathologically. This was a limiting factor for this study; in our study, we functionally and histopathologically confirmed the protective, regenerative, and anti-inflammatory effects of MSCs and migration and homing of MSCs to the site of injury in a BAC induced DES model in rats. There are studies showing inhibitory effects of intravenously administered MSCs on inflammation and therapeutic effects on experimental and clinical Sjögren syndrome [27, 28]. Topically applied MSCs can penetrate into conjunctival epithelium and meibomian glands and could decrease inflammation by their anti-inflammatory effects. This may be mediated by paracrine effects, differentiation, or transdifferentiation of topically transferred MSCs to goblet cells or glandular cells, immunomodulatory effects of transferred MSCs, or stimulation of repair mechanisms of damaged goblet cells of conjunctiva. In our study, topically applied BrdU labeled MSCs infiltration was mostly observed in basal portions of conjunctival epithelium and meibomian glands. This could be explained by homing characteristics of MSCs to injury site. Our study is the first to show conjunctival epithelium and meibomian gland migrations of BrdU labeled MSCs and homing of topically applied BrdU labeled MSCs to site of injury. This study may open new insights for further translational studies.

One of the main limitations of our study was that BAC induced DES model does not entirely represent DES commonly seen in clinics, because DES is complex disorder that has multiple complex pathophysiological pathways. BAC induced DES model has its unique characteristics and limitations. This model could be suitable especially for the study of inflammatory type of DES [10]. We choose this type of DES model which has pure inflammatory characteristics in order to show immunomodulatory and anti-inflammatory effects of MSCs. Numerous animal models have been described for DES, but after establishing the DES in these models, animals tend to recover within two weeks. Li et al. have demonstrated that topical application of 0.1% BAC for a period of 2–4 weeks could successfully produce a satisfactory and applicable rabbit model of DES. Lin et al. have demonstrated that 0.1% BAC was an appropriate mouse model for the study of inflammatory DES. Stability of BAC induced DES model was confirmed in rabbits, but we do not know whether 0.2% BAC induced DES is a stable model for rats. This point should be further clarified. Another limitation for this study was the small sample size and short duration of the study. Longer duration of the study with more animals would be useful and relevant to see extended effects of MSCs and effects of BAC in the control group in the long term.

In conclusion, DES could be effectively treated by topical application of MSCs. Topical application of MSCs can lead to an increase in aqueous tear volume and improvement in ocular surface evaluation tests in DES rats. Topical application of MSCs is a safe, easy applicable method and may easily be used for clinical research studies. Further studies with larger sample size and on different type of DES models are needed to clarify the exact mechanism of how topically applied MSCs show therapeutic effects on DES.
